# Impact of Management on Avian Communities in the Scottish Highlands

**DOI:** 10.1371/journal.pone.0155473

**Published:** 2016-05-19

**Authors:** Scott Newey, Karen Mustin, Rosalind Bryce, Debbie Fielding, Steve Redpath, Nils Bunnefeld, Bronwen Daniel, R. Justin Irvine

**Affiliations:** 1 The James Hutton Institute, Aberdeen, United Kingdom; 2 Hedmark University College, Campus Evenstad, Koppang, Norway; 3 Centre of Excellence for Environmental Decisions, School of Biological Sciences, University of Queensland, Brisbane, Australia; 4 Aberdeen Centre for Environmental Sustainability, Institute of Biological & Environmental Sciences, Aberdeen University, Aberdeen, United Kingdom; 5 The Centre for Mountain Studies, Perth College University of the Highlands & Island, Perth, United Kingdom; 6 Department of Life Sciences, Imperial College London, Silwood Park, Ascot, United Kingdom; 7 School of Natural Sciences, University of Stirling, Stirling, United Kingdom; Centre for Ecological and Evolutionary Studies, NETHERLANDS

## Abstract

The protection of biodiversity is a key national and international policy objective. While protected areas provide one approach, a major challenge lies in understanding how the conservation of biodiversity can be achieved in the context of multiple land management objectives in the wider countryside. Here we analyse metrics of bird diversity in the Scottish uplands in relation to land management types and explore how bird species composition varies in relation to land managed for grazing, hunting and conservation. Birds were surveyed on the heather moorland areas of 26 different landholdings in Scotland. The results indicate that, in relation to dominant management type, the composition of bird species varies but measures of diversity and species richness do not. Intensive management for grouse shooting affects the occurrence, absolute and relative abundance of bird species. While less intensive forms of land management appear to only affect the relative abundance of species, though extensive sheep grazing appears to have little effect on avian community composition. Therefore enhanced biodiversity at the landscape level is likely to be achieved by maintaining heterogeneity in land management among land management units. This result should be taken into account when developing policies that consider how to achieve enhanced biodiversity outside protected areas, in the context of other legitimate land-uses.

## Introduction

Halting and reversing the current and rapid loss of biodiversity is a major contemporary challenge for society [1,2]. Biological diversity is important both for its intrinsic value and because of its fundamental role in providing ecosystem services and benefits upon which we all depend [2–6]. While the use of protected areas remains a cornerstone of biodiversity conservation (hereafter conservation), the majority of land lies outside protected areas, and it is increasingly acknowledged that conservation needs to be achieved in the wider countryside [7]. Most of this land is multifunctional so conservation actions need to be integrated with other land-uses that provide livelihoods, recreation and cultural benefits [2,8,9]. Land use and land use change are key drivers of biodiversity change [4], and understanding this relationship at the landscape scale is essential [10,11] if we are to achieve internationally recognised policy goals at more local levels [2,6].

Pressure on landscapes to provide multiple benefits for a range of interests mean that neighbouring land owners and managers often pursue diverse and contrasting management objectives. For example, the Scottish uplands are largely privately owned and are dominated by moorland managed for recreational shooting but are also used for sheep grazing, commercial forestry and, increasingly, conservation management [12–14]. An estimated 4.4 million hectares, or 50% of the Scottish land area, is directly or indirectly managed for or influenced by recreational shooting—typically red grouse *Lagopus lagopus scotica* shooting, and red deer *Cervus elaphus* and roe deer *Capreolus capreolus* stalking [14,15]. Upland areas of Scotland are also associated with extensive livestock grazing, primarily by sheep, for meat production and as a habitat management tool. A substantial area of land, approximately 250,000 ha, is owned or managed by environmental non-government organisations, and a further 574,000 ha are managed by government agencies as National Nature Reserves or for commercial forestry. However, the Scottish uplands are also recognised as an internationally important ecosystem containing most of the heather-dominated moorland in the UK, and harbouring large areas of heath and mire plant communities endemic to the British Isles as well as a unique assemblage of breeding birds, eight of which are listed in Annex 1 of the European Commission Birds Directive 70/409/EEC [16].

The management of these upland areas typically differs in the extent to which four main management practices are carried out: rotational burning of heather, predator control, grazing management and conservation practices. Managing moorland for red grouse typically involves burning patches of heather (*Calluna vulgaris* and *Erica* spp) in rotation to create a mosaic of different aged stands to provide young shoots suitable as food for red grouse and older stands suitable for nesting and cover. Burning may also be employed to improve grazing for sheep. Studies have shown that rotational burning may favour some species of upland birds including curlew *Numenius arquata*, golden plover *Pluvialis apricaria* and lapwing *Vanellus vanellus* [17] but can also be detrimental to other bird species: meadow pipit *Anthus pratensis*, crow *Corvus corone* and wheatear *Oenanthe oenanthe* [17,18].

The management of predators to increase survival and/or reproduction of game and conservation species is widespread [19–23]. In the UK legal control of avian (crows *Corvus sp and Cornix spp*.), and mammalian (red fox *Vulpes vulpes*, stoat *Mustela erminea* and weasel *Mustela nivalis)* predators is a common practice on both upland estates managed for recreational shooting and land managed by conservation organisations [21].

Grazing by livestock and wild herbivores is a major driver of land-use and habitat change worldwide and can maintain or suppress vegetation diversity and associated communities depending on habitat, relative abundance of different grazers and the level of grazing [24–27]. Maintaining densities of sheep at commercially viable densities and deer at densities suitable for deer stalking can be associated with habitat degradation through over grazing and trampling [26,28].

Management objectives of environmental NGOs and government agencies will focus on the conservation of species, habits or landscapes. Management practices and intensity will vary depending on the organisation, but may include predator control, habitat management, population control of grazers, and rotational burning.

Whilst it is straightforward to identify the activities associated with particular management objectives described above, most land owners have multiple objectives and use a variety of management practices to varying degrees depending on their main objective and geography. Therefore these broad categories can be considered as being representative of the spectrum of interventions practised by land managers in the Scottish uplands.

Birds are commonly used as an indicator of biodiversity and a number of studies have looked at the effects of different moorland management objectives and practices on the occurrence and abundance of specific upland bird species. There is less information on how the different management objectives and practices affect diversity and community composition (but see; [29]) although there is some work on the effects of management on plant diversity in these habitats [27]. In this paper we investigate how the dominant management objectives and practices on a number of heather dominated moorland sites across the Scottish uplands influence avian species richness, diversity and community composition in order to investigate landscape-scale effects of management on biodiversity and the consequences for conservation policy.

## Materials and Methods

### Data collection

Twenty six upland estates (landholdings), representing different management objectives, on mainland Scotland were surveyed for breeding birds in spring-summer 2010 (Table 1). Selected estates included those previously surveyed for plant diversity, grazing, and the extent of prescribed burning [27,30]. These were chosen to represent the geographic range in the Scottish Highlands and to cover the range of different management objectives. We selected areas within estates that were representative of heather dominated moorland (in practice a heather–grass mosaic) and, so far as was practical, we selected sites within these areas that had similar aspect and slope. We also ensured there was a reasonable buffer of moorland between these sites and other habitats such as woodland so that the influence on species richness and composition from woodland was minimised. We interviewed the estate owner or head game keeper to identify the management objective(s) of the estate and whether legal avian and/or mammalian predator control was carried out. We quantified the extent of heather burning by assessing the percentage of ground showing the characteristic vegetation patterns produced by rotational burning of vegetation [31] using GoogleEarth™ aerial imagery for each 1 km^2^ bird survey square (imagery accessed December 2010; images used dated from January 2004 –January 2010) [30]. While images span a six year time period, the characteristic burn patterns are thought to persist for 10–15 years post-burning, and that estates aim to maintain a relatively constant proportion of different aged stands of heather means that imagery data is likely to be representative of management over decadal time periods [30,31]. Habitat diversity might be expected to vary with area and management objectives and influence species diversity [32,33]. We therefore used the Land Cover Map 2007 [34] to estimate the proportion of habitats within each estate and used these proportions to estimate an index of habitat diversity using the Shannon-Wiener index.

**Table 1:** Summary of management objectives and activities associated with each estate.

Dominant management objective	Total number of sites	Number where this is the sole objective	Number practicing MPC	Number practicing APC	Number practicing prescribed burning
Grouse shooting	10	1	10	9	10
Deer stalking	12	5	12	9	12
Conservation	7	5	5	4	6
Sheep grazing	11	5	8	6	9

MPC = mammal predator control, APC = avian predator control.

For each estate, bird surveys were carried out on one to four (median = 3) 1 km^2^ areas using a modified version of the Breeding Bird Survey [35]. Two parallel equidistant transects (500 m apart; total length 2 km) were traversed on foot at a constant slow walking speed by a single observer. All bird surveys were carried out within four hours after dawn, during the time of peak bird activity. Surveys were avoided under conditions of poor visibility and persistent rain. Birds within 250 m of the transect line were identified to species and counted. Care was taken to avoid double-counting the same birds. Each area was surveyed twice; once in April-May and again in June-July to increase the probability of detecting all species breeding and utilising heather moorland.

**Fig 1:**
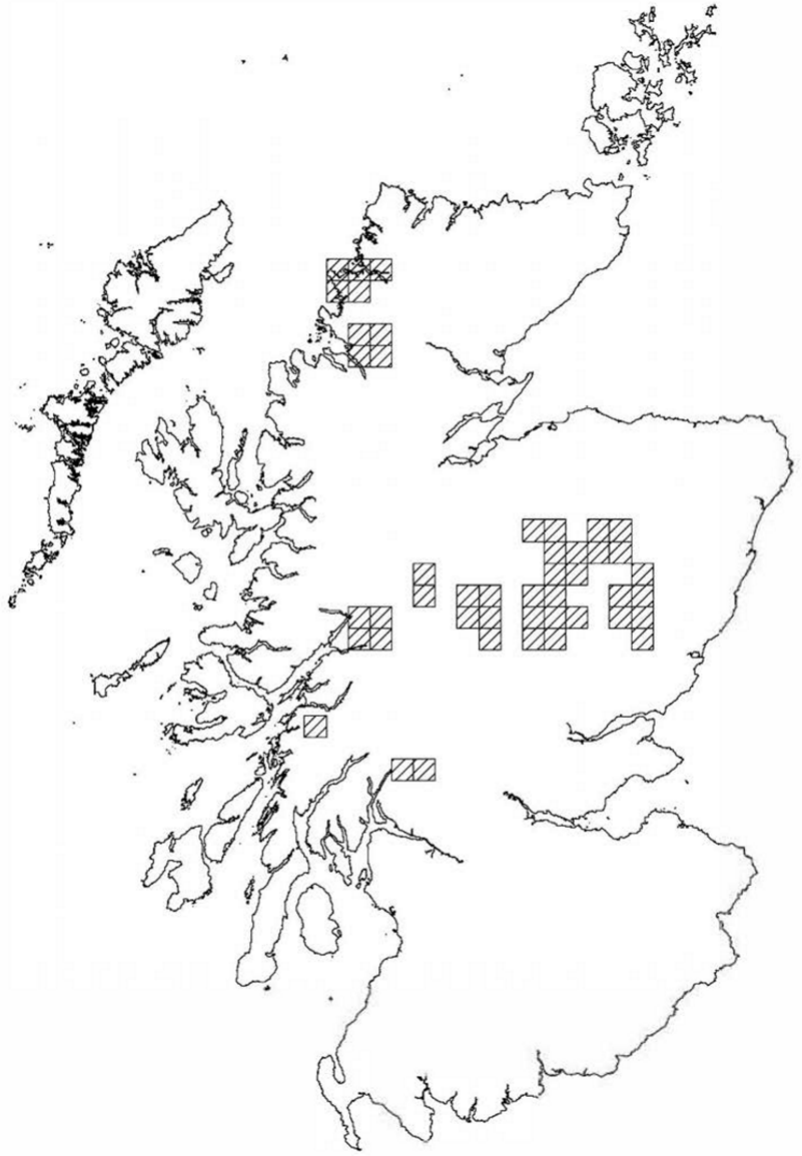
Map showing the 10 km blocks within which the study sites were located.

Study sites were located on private land subject on the condition that the landowner and land holding remain anonymous. Therefore estate locations cannot be provided but, Fig 1 shows the approximate location of sites within Scotland, all of which were in the Highlands,

### Data analysis

The aim of these analyses was to relate avian species diversity and community composition to the dominant management objectives and practices on heather-dominated moorland sites. Interviews indicated that the management objectives were applicable at the level of the estate not at the level of the survey square so all analyses were carried out at the estate scale. Most estates (62%) categorised themselves as having one dominant management objective, though 10 of 26 were managed for more than one dominant management objective prohibiting any direct comparison of different management objectives. All analyses were therefore carried out by comparing estates with a particular dominant management objective or activity with those estates with other objectives, for example; estates which included grouse shooting as a management objective are compared to those estates that did not include grouse shooting as a dominant management objective.

Estimated percentage of heather burnt and habitat diversity were compared between estates using linear models with an identity link. To normalise model residuals we used the natural logarithm of percentage area burnt (plus one), and the natural logarithm of estate habitat diversity as the response variable. The differences in the proportion of estates carrying out different forms of predator control were compared using an Exact Binomial Test.

Bird survey data were pooled for all of the survey squares on each estate. Abundance estimates were taken from the highest count of the two surveys. Abundance data were used to estimate two measures of Alpha diversity: species richness (number of species) and species diversity (Shannon-Wiener index) [36,37]. The bird survey data were also used to analyse the variation in community composition.

### Alpha diversity analysis

Visual inspection of variograms (R-package nlme) [38] suggested no evidence of spatial autocorrelation for species richness or species diversity. We therefore used a Generalised Linear Model with Poisson error and log link function to investigate how bird species richness varied with management objectives (grouse shooting, deer stalking, sheep grazing or conservation), management practices (prescribed burning, and predator control; mammal control only and combined mammal and avian control), and estate level habitat diversity. We investigated species diversity measured by the Shannon-Wiener index using a linear model with the same explanatory variables. Examination of model residuals suggested that estimates of Shannon-Wiener index for both avian diversity and estate habitat diversity gave rise to non-normal residuals and therefore we analysed the natural logarithm of these response variables. In Scotland, climate, habitat and land management show geographic trends with the cooler, drier east of the country tending to support grouse moor management while the warmer and wetter west of the country tends to support more sheep production and deer stalking. These climatic gradients have been shown to influence upland avian diversity [18] and we controlled for this in the model by fitting latitude and longitude as covariates.

We aim to explain the effect of different land management objectives and practices on avian diversity and therefore adopt a null-hypothesis testing framework to test the significance and estimate effect sizes of explanatory variables [39] rather than an information theoretical approach more applicable to predictive modelling [40]. Explanatory variables in this study are not always mutually exclusive or necessarily independent. For example, some estates had more than one dominant management objective, and prescribed burning is strongly associated with grouse moor management. To account for collinearity of explanatory variables we limit our analysis to assessing one explanatory factor or variable of interest at a time. We tested each explanatory variable by adding each term individually to a statistical model containing only latitude and longitude and tested for significance (p<0.05) using F- or X^2^- tests. All analyses were carried out in R 3.2.3 [41].

### Community composition analysis

In this study we explore relationships between community composition and particular factors (management objective) and environmental variables of interest and therefore use unconstrained ordination. We used Non-Metric Multi-Dimensional Scaling (NMDS) [42,43] and permutation tests to assess the effects of dominant management objective, avian predator control, avian and mammalian predator control, prescribed burning, and habitat diversity on species composition using the ‘metaMDS()’ and ‘envfit()’ function in the R-package ‘vegan’ [44]. Different approaches to data treatment and ordination can reveal different aspects of community composition, for example; ordination methods based on presence/absence compared to abundance data, or differences in how methods treat joint absences can have profound effects on ordination results [43]. Here we use three different methods of treating community data to explore different aspects of the observed communities; i) analysis based on the Bray-Curtis dissimilarity index using square root transformed species abundance data which emphasises differences in community composition and relative abundance, ii) analysis of Euclidean distance based on raw abundance data which emphasises differences in raw abundance of species and includes joint absences, and iii) analysis of presence/absence data using Raup-Curtis measure which includes joints absences. This method accounts to some extent for differences in alpha diversity (species richness) which can otherwise skew comparison of communities with different species richness helping to highlight differences in the occurrence of species [43].

## Results

### Management practices

Four dominant management objectives emerged from interviews with land owners: grouse shooting; deer stalking; sheep grazing; and conservation (Table 1). While the majority of estates were managed for one dominant objective (16/26), ten estates classified themselves as being managed for multiple dominant objectives (Table 1). The most common management activity was muirburn (23/26 estates). Estates managed for grouse shooting and deer stalking had a significantly higher percentage of burnt ground compared to estates that did not include these management objectives (Table 2A). Although the mean percentage of burnt ground was lower on estates that included conservation and sheep grazing in their dominant management objectives this differences was not significant (Table 2A). Despite differences in the percentage of burnt ground estate level habitat diversity did not vary consistently with the dominant estate management objective (Table 2B).

**Table 2:** a). The occurrence and intensity of rotational burning for each dominant management objective. b). Estate habitat diversity (Shannon Index) described for each dominant management objective.

Dominant Management Objective	No (Mean, SD, range)	Yes (Mean, SD, range)	Estimate (SE)
2(a). Percentage burn			
Grouse shooting	4.7% (6.57, 0.0–17.3)	14.6% (13.17, 3.9–40.7)	1.29 (0.38) F_1,24_ = 11.3, P = 0.003
Deer stalking	6.2% (11.35, 0.0–40.7)	11.3% (9.40, 0.7–36.2)	1.05 (0.40) F_1,24_ = 7.0, P = 0.014
Conservation	9.8% (11.52, 0.0–40.7)	5.2% (7.21, 0.0–17.3)	0.56 (0.50) F_1,24_ = 1.29, P = 0.27
Sheep grazing	11.1% (12.66, 0.0–40.7)	5.0% (5.7, 0.0–15.0)	-0.62 (0.44) F_1,24_ = 1.98, P = 0.17
2(b). Estate habitat diversity			
Grouse shooting	0.816 (0.438, 0.001–1.465)	0.936 (0.316, 0.468–1.451)	0.66 (0.61) F_1,24_ = 1.17, P = 0.29
Deer stalking	0.806 (0.470, 0.001–1.465)	0.928 (0.285, 0.460–1.319)	0.75 (0.60) F_1,24_ = 1.58, P = 0.22
Conservation	0.867 (0.411, 0.001–1.451)	0.849 (0.370, 0.468–1.465)	0.39 (0.68) F_1,24_ = 0.33, P = 0.57
Sheep grazing	0.960 (0.308, 0.460–0.465)	0.729 (0.469, 0.001–1.444)	-1.028 (0.58) F_1,24_ = 3.14, P = 0.089

Yes/No indicates the estates that included/did undertake a particular dominant management objective. Estimate (with Standard Error) shows the estimated effect size of each objective.

Twenty-one of the 26 estates carried out mammalian predator control and 16 carried out avian predator control; all estates that controlled avian predators also controlled mammalian predators (Table 1). All estates with grouse shooting or deer stalking as dominant management objectives carried mammalian predator control (Table 1). Mammalian predator control was therefore significantly more prevalent among estates with grouse shooting (p< 0.01) or deer stalking (p< 0.01). Although the number of estates carrying out mammalian predator control was, when compared to corresponding estates not carrying out conservation or sheep production, greater among estates with conservation or sheep production as dominant management objectives the differences were not significant (Table 1; conservation; p = 0.22, sheep production; p = 0.11). Avian predator control was significantly more prevalent only on estates that included grouse shooting as a dominant management objective (Table 1; red grouse shooting; p<0.05, deer stalking; p = 0.07, conservation; p = 0.5, sheep production; p = 0.5),

### Avian diversity and community composition

In total, 58 species of bird were recorded during surveys (S1 Table). The upland bird communities were dominated by 3 species accounting for 77% of all observations; meadow pipit (51%), red grouse (15%) and skylark *Alauda arvensis* (11%). No other single species accounted for more than 5% of the observed community.

#### Species richness

There was no significant effect of any management objective or practice, or percentage of burnt ground on avian species richness (Table 3). Estimates of species diversity are associated with large variation and a high degree of uncertainty, for example; on average deer stalking estates supported three fewer species, representing a relatively large effect size of -20%, compared to the rest of the estates however the range of species richness for deer stalking estates range from 0–6 representing a high degree of uncertainty in the estimated effect size (Table 3). Avian species richness increased significantly with estate habitat diversity (Table 3).

**Table 3:** The effects of dominant management objectives and management activities on avian species richness and diversity.

	Species Richness			Species Diversity		
Management (n)	No (Mean (SD, range))	Yes (Mean (SD, range))	Estimate (SE)	No (Mean (SD, range))	Yes (Mean (SD, range))	Estimate (SE)
Deer (12)	15 (6, 8–27)	11.7 (3.2, 7–18)	-0.22 (0.12) Χ^2^_1_ = 3.42, P = 0.064	1.56 (0.37, 1.19–2.47)	1.45 (0.27, 1.03–1.88)	-0.050 (0.084) F_1,23_ = 0.35, P = 0.56
Conservation (7)	13.5 (4.8, 8–27)	13.1 (6.2, 7–25)	0.0001 (0.027) Χ^2^_1_ = 0.0001, P = 0.99	1.56 (0.35, 1.03–2.47)	1.38 (0.23, 1.16–1.78)	-0.086 (0.088) F_1,23_ = 0.95, P = 0.34
Grouse (11)	14.2 (5.9, 8–27)	12.2 (3.4, 7–18)	0.14 (0.19) Χ^2^_1_ = 0.531, P = 0.470	1.54 (0.38, 1.03–2.47)	1.45 (0.21, 1.13–1.79)	0.157 (0.126) F_1,23_ = 1.56, P = 0.23
Sheep (11)	12.9 (4.6, 8–25)	14.1 (5.9, 7–27)	0.06 (0.11) Χ^2^_1_ = 0.26, P = 0.61	1.46 (0.27, 1.03–1.88)	1.57 (0.39, 1.16–2.47)	-0.060 (0.078) F_1,23_ = 0.58, P = 0.45
APC+MPC (16)	13.9 (6.2, 8–27)	13.1 (4.5, 7–25)	0.11 (0.14) Χ^2^_1_ = 0.694, P = 0.4	1.58 (0.44, 1.03–2.47)	1.47 (0.23, 1.13–1.88)	0.01 (0.10) F_1,23_ = 0.01, P = 0.92
Habitat Diversity (na)	na	na	0.09 (0.04) Χ^2^_1_ = 4.78, P = 0.029*	na	na	0.041 (0.03) F_1,23_ = 2.13, P = 0.16
%Burn (na)	na	na	-0.003 (0.007) Χ^2^_1_ = 0.152, P = 0.7	na	na	0.003 (0.005) F_1,23_ = 0.52, P = 0.48

Yes/No indicates the estates that included/did not include a particular objective or activity. n = number of estates/landholdings. Estimate (with Standard Error) shows the estimated effect size of each objective or activity.

* denotes a statistically significant effect.

APC = avian predator control.

MPC = mammalian predator control.

%Burn = Estimated percentage of ground showing signs of prescribed burning.

na = not applicable.

#### Species diversity

Differences in Shannon-Wiener index of species diversity, between management objective and practices were small and subject to relatively large variation and there was no significant effect of any management objective or practice on avian diversity (Table 3). Avian diversity increased non-significantly with estate level habitat diversity (Table 3).

#### Community composition

**Fig 2:**
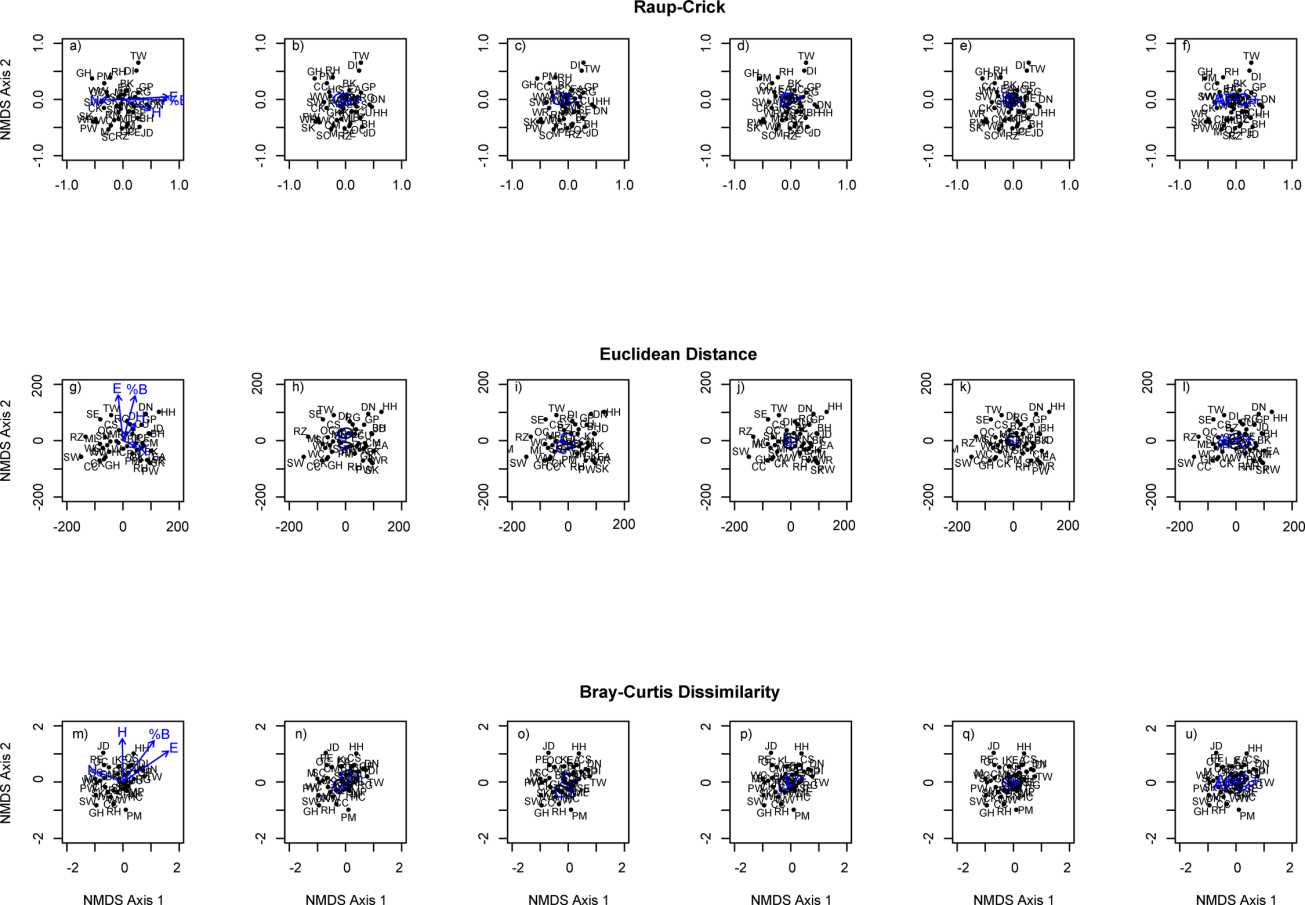
Ordination plots of Raup-Crick (a-f), Euclindean Distance g-l), and Bray Curtis Dissimilarity (m-u) with 95% Standard Error centroids showing species associated with dominant management and management activities (e.g. G+ denotes estates managed for grouse and G- indicates the rest of the estates). Species are coded following British Trust for Ornithology survey codes; Mallard—MA; Tufted Duck—TU; Red Grouse—RG; Ptarmigan—PM; Black Grouse—BK; Pheasant—PH; Red-throated Diver—RH; Cormorant—CA; Grey Heron—H.; Hen Harrier—HH; Buzzard—BZ; Golden Eagle—EA; Kestrel—K.; Merlin—ML; Peregrine—PE; Moorhen—MH; Oystercatcher—OC; Golden Plover—GP; Lapwing—L.; Dunlin—DN; Snipe—SN; Curlew—CU; Greenshank—GK; Common Sandpiper—CS; Black-headed Gull—BH; Common Gull—CM; Lesser Black-backed Gull—LB; Herring Gull—HG; Greater Black-backed Gull—GB; Woodpigeon—WP; Cuckoo—CK; Short-eared Owl—SE; Swallow—SL; Meadow Pipit—MP; Pied Wagtail—PW; Dipper—DI; Dunnock—D; Robin—R.; Whinchat—WC; Stonechat—SC; Wheatear—W.; Ring Ouzel—RZ; Skylark—S.; House Martin—HM; Wren—WR; Mistle Thrush—M.; Grasshopper Warbler—GH; Sedge Warbler—SW; Chiffchaff—CC; Willow Warbler—WW; Great Tit—GT; Jackdaw—JD; Carrion/Hooded Crow—HC.; Raven—RN; Chaffinch—CH; Greenfinch—GR; Siskin—SK; Twite–TW. (Larger plots are provided in S1 Fig).

There is large degree of agreement among the three ordinations used (Table 4). Ordinations based on the Bray-Curtis dissimilarity index, Euclidean distance and the Raup-Crick method reveal that latitude, percentage burnt ground and management for grouse shooting have significant effects on absolute and relative abundance of species, and community composition (Tables 4 and 5; Fig 2A, 2B, 2G, 2H, 2M and 2N). Species such as sandpiper, red grouse, golden plover, and dunlin are strongly influenced by latitude and burning, and are more likely to be prevalent on estates in the east and where there is greater percentage of burning (Table 4, Fig 2A). The absolute and relative abundance of some wader species, e.g. curlew, dunlin and sandpiper, red grouse, black-headed gull, golden eagle and buzzard are positively associated with increasing percentage of burnt ground and on more easterly estates. (Table 4, Fig 2G and 2M). While grouse shooting as a dominant management objective appears to have a strong influence on the occurrence and absolute abundance of only a few species, these estates were still associated with a distinctive avian assemblage (Tables 4 and 5, Fig 2B, 2G and 2M) characterised by wading birds such as; curlew, golden plover, and common sandpiper as well as; black-headed gull, buzzard, short-eared owl, red grouse, and meadow pipit. However, these estates were also negatively associated with corvids, merlin, and some passerine species (Table 5, Fig 2B, 2H and 2N).

**Table 4:** Results of permutation tests on the effects of management objective and management practices on the composition of avian diversity.

	Bray-Curtis^1^		Euclidean^2^		Raup-Crick^3^	
NMDS variable/factor	r^2^	P	r^2^	P	r^2^	P
Latitude (Easting)	0.35	0.01	0.40	0.01	0.37	0.01
Longitude (Northing)	0.08	0.37	0.04	0.64	0.09	0.33
Burning	0.31	0.01	0.41	0.01	0.34	0.01
Habitat diversity	0.11	0.28	0.06	0.52	0.06	0.55
Grouse	0.20	0.01	0.22	0.01	0.22	0.01
Deer	0.14	0.02	0.04	0.36	0.10	0.08
Conservation	0.15	0.02	0.10	0.07	0.11	0.05
Sheep	0.01	0.71	0.01	0.84	0.04	0.35
APC (plus MPC)	0.04	0.37	0.04	0.41	0.06	0.24

MPC = mammalian predator control, APC = avian predator control.

1. Bray-Cutis dissimilarity calculated from square root transformed abundance data

2. Euclidean distance calculated from raw abundance data

3. Raup-Crick index calculated on presence-absence data.

**Table 5:** Community composition in relation to management objectives and practices. Significant positive and negative associations are shown for each species for the management objectives, practise and variables that were identified as significant in the ordination analyses.

	Bray-Curtis Dissimilarity					Euclidean Distance			Raup-Crick		
Species	GS	DS	C	%B	Lat.	GS	%B	Lat.	GS	%B	Lat.
Red grouse (*Lagopus lagopus*)	++	++	-		+	+	++	+	+	++	++
Black grouse (*Tetrao tetrix*)	—	—	-								
Curlew (*Numenius*. *arquata*)	++	+	—	++		+	+		+		
Golden plover (*Pluvialis apricaria*)	+			+	++				+		
Greenshank (*Tringa nebularia*)	+	+	-						-		
Snipe (*Gallinago gallinago*)	+	+	—						-		
Dunlin (*Calidris alpine*)					++		+			+	
Common sandpiper (*Actitis hypoleucos*)				+		++		+	++	++	++
Buzzard (*Buteo buteo*)	++	+	—	+		++	+				
Merlin (*Falco columbarius*)	—	—	++								
Golden eagle (*Aquila chrysaetos*)				+					+		
Short-eared owl (*Asio flammeus*)	++	+	-						++		
Black-headed gull (*Chroicocephalus ridibundus*)	++	+		++							
Common gull (*Larus canus*)	+		-								
Crow (*Corvus corone/cornix)*	-	-	++			—			-		
Raven (*Corvus corax*)	—	—	++			-					
Ring ouzel (*Turdus torquatus*)	—	—	-								
Meadow pipit (*Anthus pratensis)*	—	—	+			-			—		
Skylark (*Alauda arvensis)*	—	—	++			—			—		
Wheatear (*O*. *oenanth)*	—	—	+			—			-		
Willow warbler (*Phylloscopus trochilus*)	-	-	+								
Wren (*Troglodytes troglodytes*)	-	-	++								
Chaffinch (*Phylloscopus collybita*)			+								
Cuckoo (*Cuculus canorus*)			+								
Dipper (*Cinclus cinclus*)					++	+	++				

GS–Grouse shooting, DS–Deer stalking, C–Conservation, %B–Percentage burnt ground, Lat.–Latitude (Easting). For factors (dominant management objectives, Predator control) positive associations are defined as those species lying within (++) or close to (+) the 95% ‘Yes’ 95% Standard Error centroid, and negative associations are defined as those species lying within (—) or close to (-) the 95% ‘No’ 95% Standard Error centroid in the NMDS ordination plots. For continuous variables (latitude and percentage ground burnt) positive associations are classed as those species lying on (++) close to (+) the arrow in the NMDS ordination plots.

Conservation as a dominant management objective had clear and statistically significant effects on the relative abundance of species, though the effect on absolute abundance and occurrence is less clear (Tables 4 and 5, Fig 2C, 2I and 2O). These estates tended to be positively associated with passerine species, corvids and merlin; and negatively associated with red and black grouse, some wading birds, buzzard, short-eared owl, common gull and ring ouzel (Table 5, Fig 2O). However, whether an estate was managed for deer stalking, sheep grazing or not had no significant effect on community composition (Table 4, Fig 2D, 2E, 2I, 2J, 2O and 2P). There was no significant effect on avian community composition of predator control, estate habitat diversity or latitude (Table 4, Fig 2F, 2L and 2U).

## Discussion

In this study we investigated how species richness, diversity and community composition vary in relation to management objectives and practices. We found no significant effect of any management activity on overall avian diversity, however, some species and assemblages are more strongly associated with certain management types. This implies that maintaining diversity in land management at the landscape scale, at least among the management types we investigated, may help to maximise biological diversity in the wider countryside.

Outside protected areas, land is managed to achieve a range of social and economic benefits and deliver food and water security, recreation opportunities and carbon storage [45]. In Scotland, as elsewhere, government policy aims to secure these ecosystem services, reduce and mitigate the causes and consequences of climate change whilst also promoting sustainable economic growth. However, achieving this with finite land whilst safeguarding the biodiversity that underpin these services is a major scientific, societal and policy challenge [45]. Conserving biodiversity and developing the green infrastructure necessary for conservation resilience depends on how well these objectives can be integrated and reconciled with the objectives of other legitimate land uses [8,9].

Our analysis of community composition provides insights as to how different management regimes and practices favour some species over others. Management of estates for red grouse shooting, characterised by intensive management (rotational burning and predator control) has a strong influence on the prevalence and abundance of species found on these estates relative to estates not managed for grouse shooting. Whereas avian communities associated with estates managed for conservation and deer stalking only differed significantly in relative abundance rather than in the presence or absence of species or their absolute abundance. These results suggests that more intensive management may be beneficial for certain species, such as some wading birds, but may be less so for other species including many passerines, and that management effects the relative abundance of species rather than their occurrence per se or absolute abundance. This is further supported by the positive association of some wading birds with higher levels of prescribed burning. This confirms earlier studies that report higher abundances of some wader species on heather moorland where grouse moor management is a dominant objective [13,17] whereas the heather-grass mosaic that results from this practice has been shown to be less favourable for some passerine species [18]. This may have consequences for predators such as hen harriers *Circus cyaneus* [18] which are partly dependant on meadow pipits. In contrast, our results show that passerines such as skylark, whinchat *Saxicola rubetra* and meadow pipit are more strongly associated with landholdings not managed for red grouse.

Although species richness was linked with habitat diversity in our study, it was not affected by management. The ordination results presented here also suggest that management influences abundance rather than prevalence and may indicate that species presence is more strongly influenced by factors other than those measured in this study, for example specific habitat requirements such as water bodies for red throated diver (*Gavia stellata*) and riparian willow (*Salix sp*.*)* scrub in the case of willow warbler and chiffchaff (*Phylloscopus collybita*). This may also offer some explanation for the apparently low species richness and diversity observed on conservation landholdings where concerted management for conservation might be expected to support greater diversity. Although we selected sites on conservation focussed landholdings that were comparable with the heather moorland on the other study sites, conservation land holdings in the Scottish uplands often include areas not far from our study sites that are managed for conservation of particularly vulnerable species or groups such as woodland grouse or water birds in habitats not included in this study (e.g. native woodland regeneration). This affect may be represented in the ordination analysis which, for example, show that woodland species such as chaffinch *Fringilla coelebs* and willow warbler *Phylloscopus trochilus* tend to be associated with conservation focused landholdings.

The lack of any significant relationship between avian richness and diversity, and dominant management objective at the estate scale is however surprising given the evidence that management affects the abundance of some upland species and that previous studies have found species diversity to be affected by management [13,17,20]. Although our findings come from a relatively small number of landholdings, our results suggest that habitat diversity may be more important than estate management in driving species richness in these heather dominated areas [13,17]. Additionally when we analysed the bird community composition on estates managed primarily for sheep production we did not find any difference from the other estates and only small differences from estates managed primarily for deer. This is in contrast to evidence that grazing by domestic and wild herbivores such as deer and sheep influences vegetation composition and structure [27] which has been shown to affect the foraging behaviour and reproductive success of insectivorous passerines such as the meadow pipit [24,25]. However, a lack of a significant effect in our sample may be because these estates are often characterised by a combination of mixed grazing, less predator control and reduced burning regimes compared to grouse and conservation landholdings, as well as a tendency for other management objectives to co-exist on the same landholding. These may combine to make it difficult to detect the effects of sheep grazing on the composition of upland bird communities.

The selective removal of predators for the benefit of other species might be expected to have a strong effect on populations of predators and their prey and there is good evidence in the literature to support this [20,46,47]. Whilst our results, suggest that predator control alone has no significant effect on avian species richness, diversity or community composition we only had a simple binary index indicating whether the management action took place or not and the widespread use of predator control in our sample of estates (including grouse moors and most conservation estates) might partially explain why we found no significant effect on our indices of bird diversity. Although it is possible that predator control on the estates we surveyed may not sufficiently reduce predator abundance to generate an observable effect on overall avian richness, a more quantitative measure of predator control would have allowed a more thorough assessment of its effects. In addition some predator and prey species are highly mobile making use of and affected by conditions on neighbouring areas that are themselves subject to different management objectives. Thus the local avian communities surveyed in this study are likely to be influenced not only by local management, but also management in neighbouring areas and landholdings [48,49].

The majority of estates included in this survey were managed for one dominant objective (16/26). However, 10 estates classified themselves as being managed for multiple dominant objectives, which may potentially dilute the ability of the analysis to detect the link between management objective or activity and avian diversity in this data set. The inability to disentangle possible explanatory factors is an inherent weakness of correlative studies such as ours. However it is important to investigate processes that affect biodiversity such as land management at the scale on which they act if we are to inform policies aimed at managing biodiversity in the wider countryside [50]. Thus while we acknowledge that our sample of land holdings do not represent a true random sample, complying with this criteria at a landscape scale is logistically very challenging and we argue that the estates selected provide a representative sample of land use and geographic coverage across the landscape to allow meaningful inference. A further refinement of our approach would be to investigate and account for uncertainty in detecting species. In our analysis we assume that species detectability remains constant across estates, habitats and time and we do not allow for uncertainty in detecting species or individuals. Future studies should aim to account for this uncertainty, by for example using occupancy modelling.

Each of the management objectives and practices investigated here support different, but overlapping, avian communities suggesting that diversity in land use types and objectives within a landscape can enhance the diversity of avian species across this landscape. Land-based businesses contribute hugely to Scottish and EU economies and our results suggest that management of private land for social and economic objectives is not necessarily at odds with conservation. Although some species are currently of higher priority in policy terms, this study suggests that there are potentially significant benefits from working with land managers to ensure best practice among the diverse management types in order to deliver landscape scale biodiversity in the wider countryside [51,52].

## Supporting Information

S1 Fig Large versions of all the ordination plots presented in Fig 2 provided for ease of reading. (PDF)

S1 File Estate management and species abundance data. Estate and bird abundance data used in analyses.
(CSV)

S1 Table Species List. Alphabetical list of all bird species recorded during surveys and indication of whether a species is included in the ordination plots (all species were included in the analysis, but for clarity not all species are shown in Fig 2).
(DOCX)
