# Supplementary material for: Impact of Management on Avian Communities in the Scottish Highlands
Source: PLoS One. 2016 May 19;11(5):e0155473. doi: 10.1371/journal.pone.0155473 (PMC4873258; doi:10.1371/journal.pone.0155473)
Supplement: S1 Fig — (PDF) [file pone.0155473.s001.pdf]

Raup-Crick NMDS Ordination Plots

a) Raup-Crick: Continous Variables

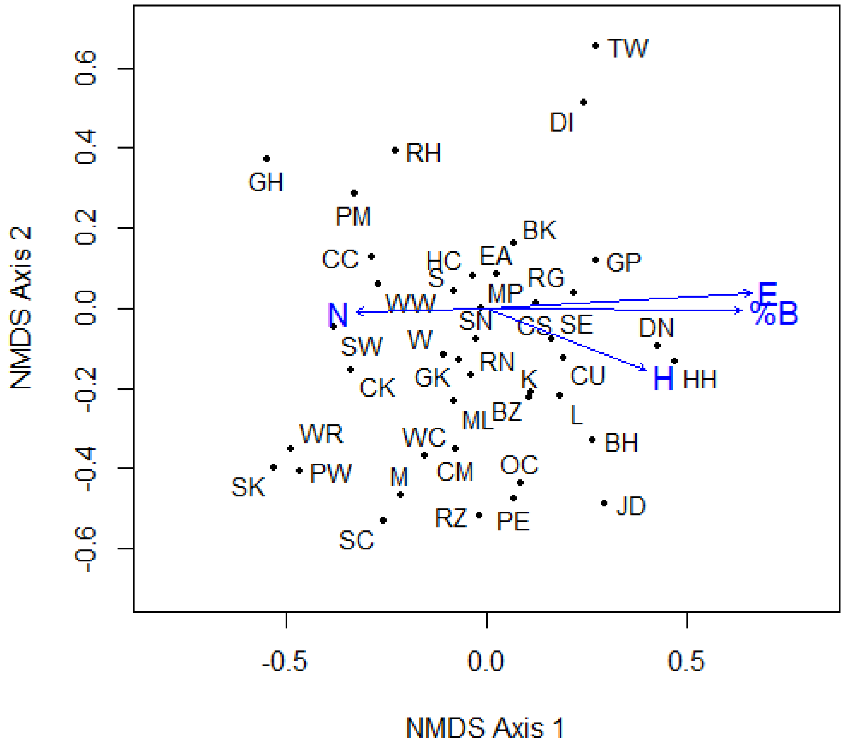

b)Raup-Crick: Grouse Shooting

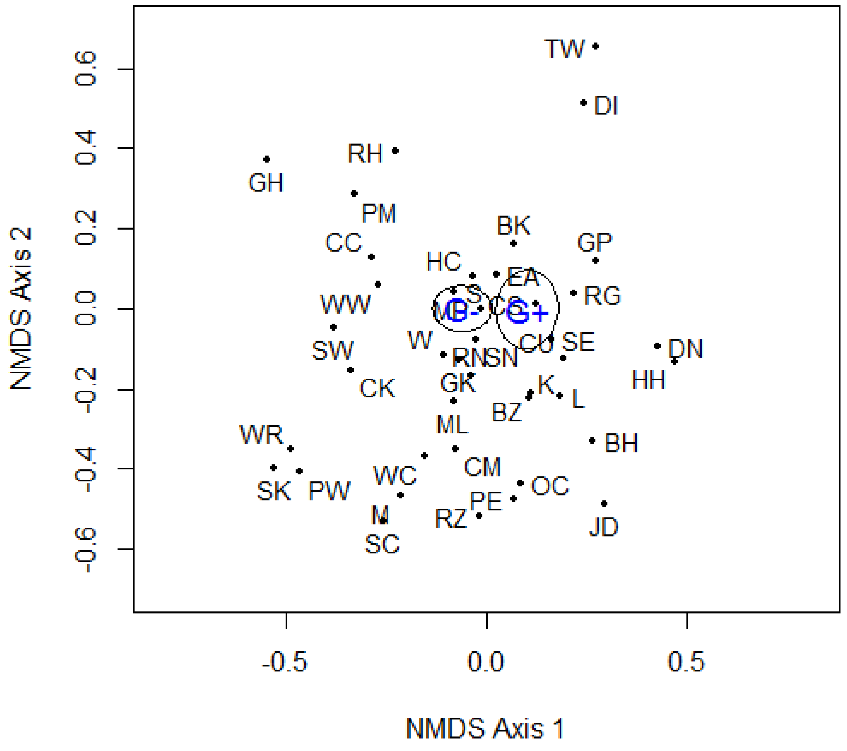

c)Raup-Crick: Conservation

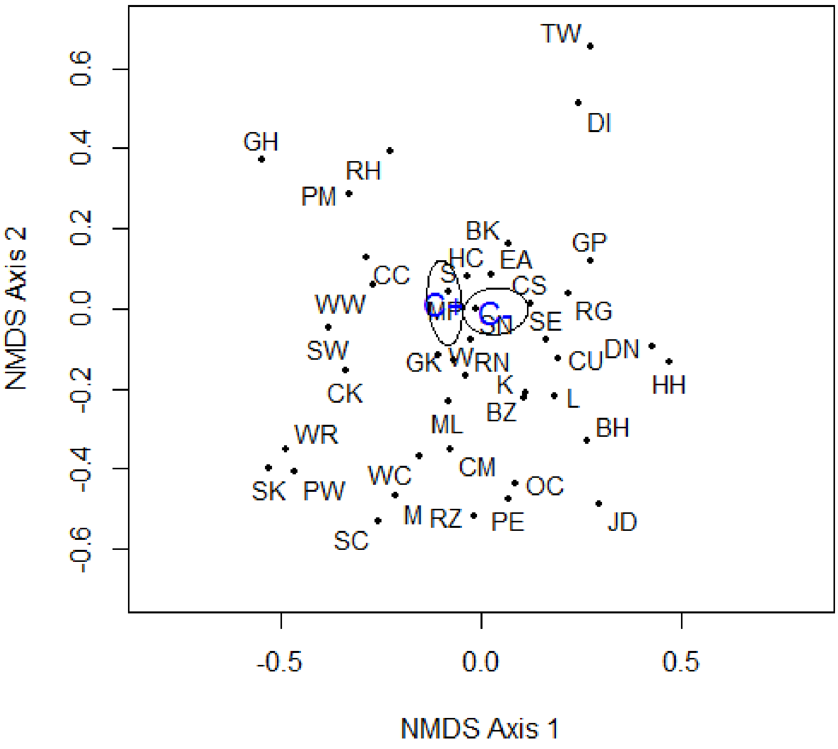

d)Raup-Crick: Deer Stalking

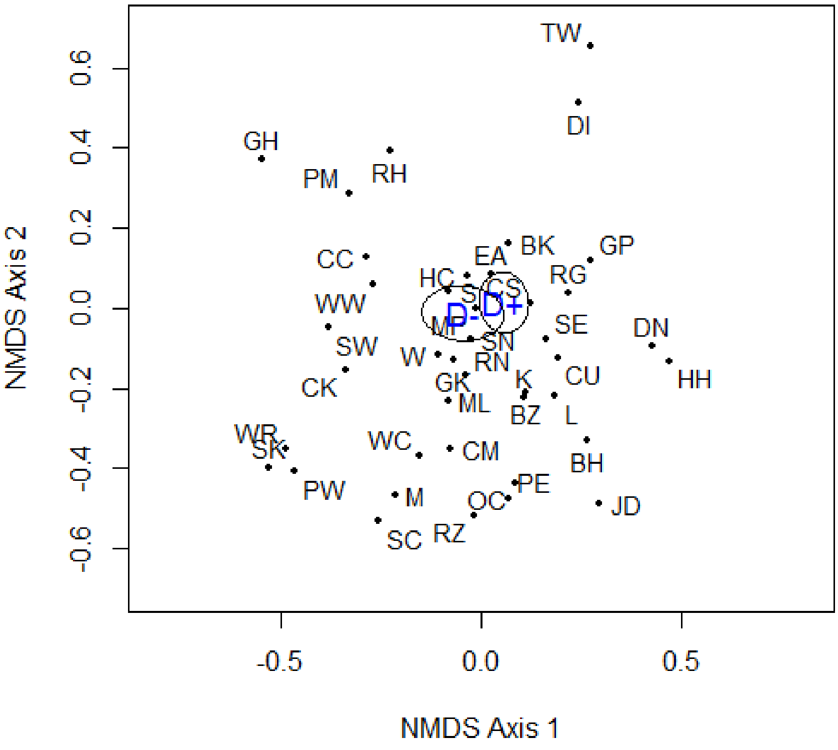

e) Raup-Crick: Sheep Grazing

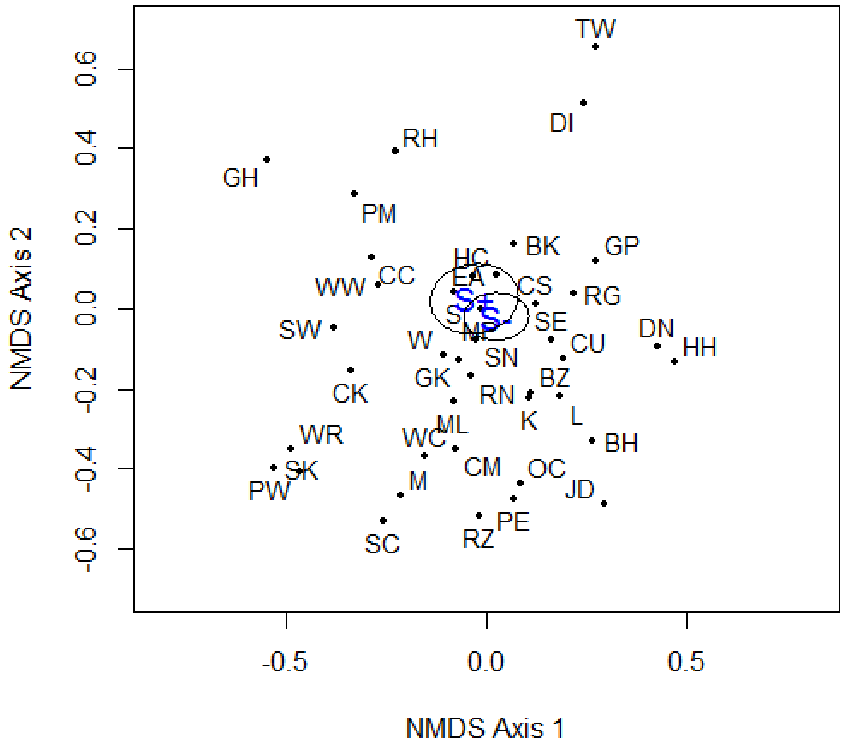

f) Raup-Crick: APC & MPC

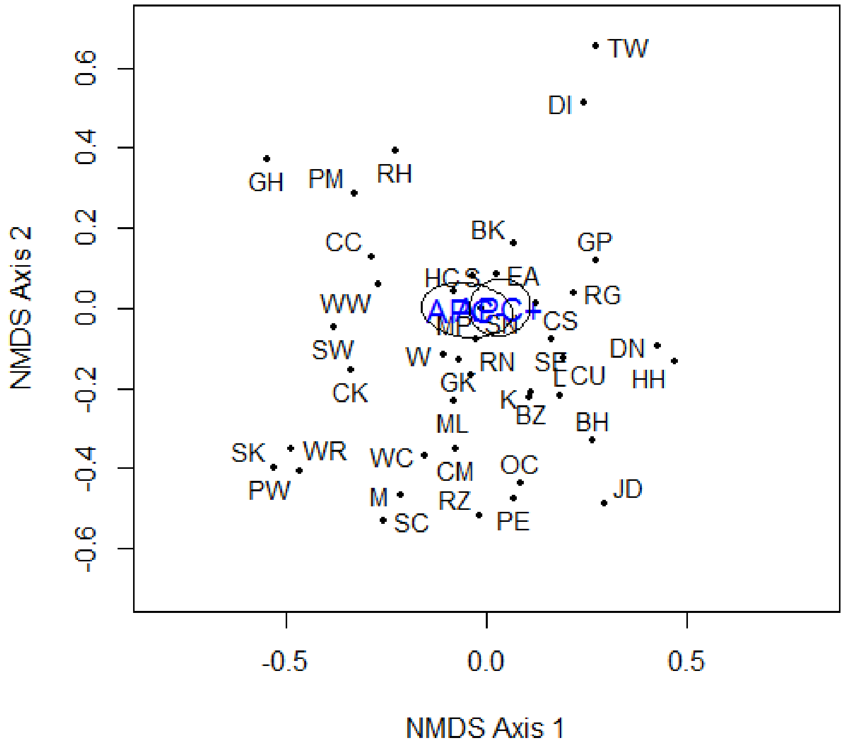

Euclidean Distance NMDS Ordination Plots

g)Euclidean Distance: Continous Variables

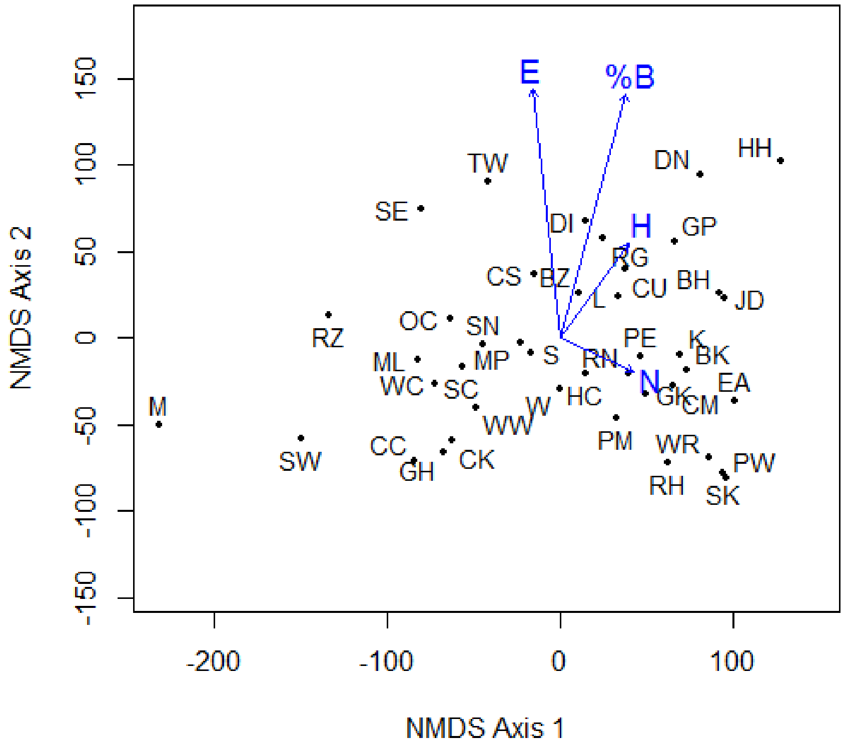

h)Euclidean Distance: Grouse Shooting

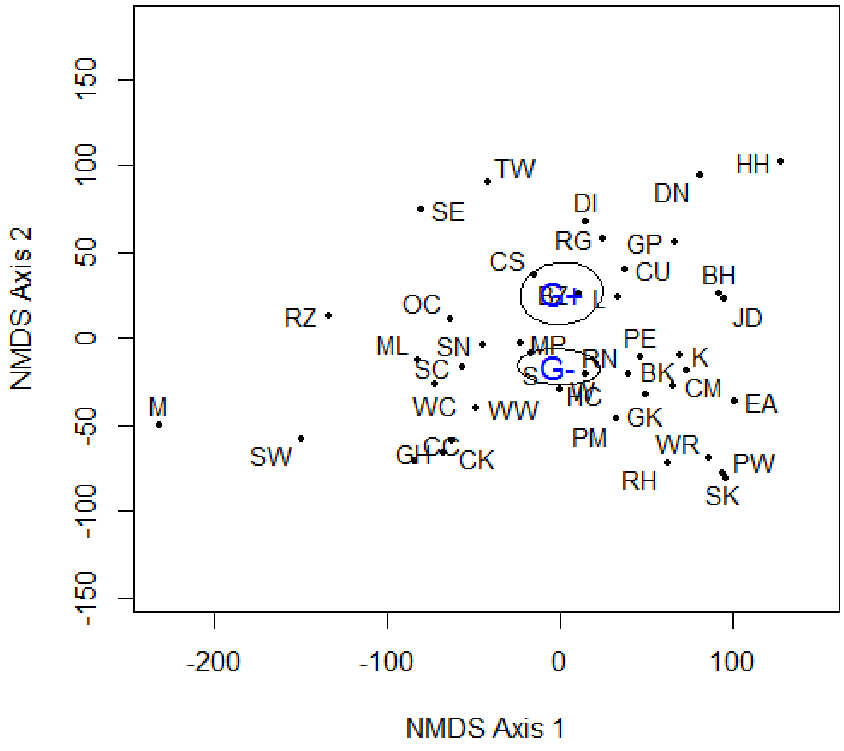

i)Euclidean Distance: Conservation

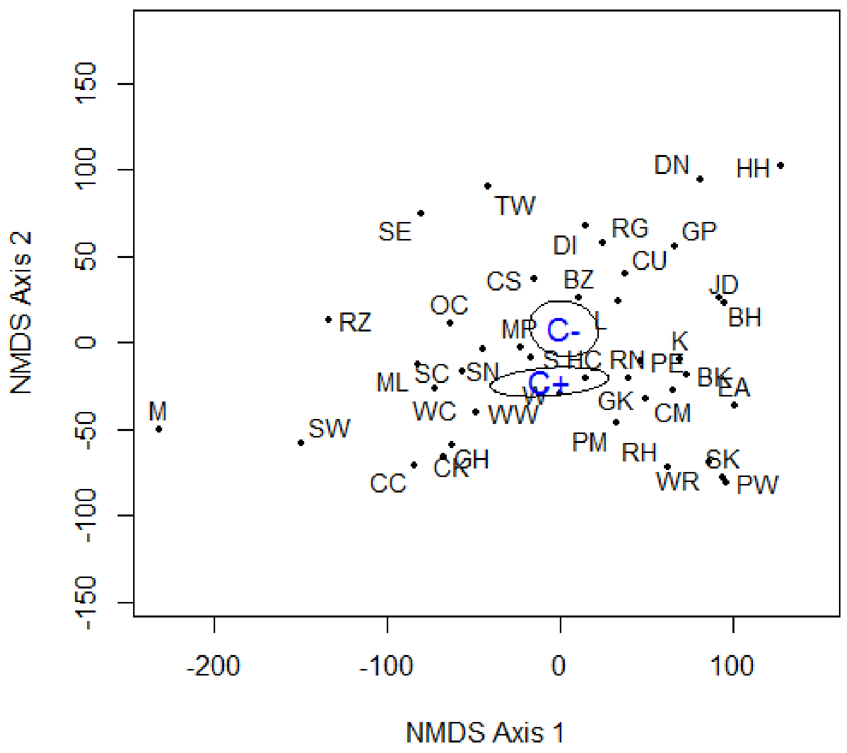

j)Euclidean Distance: Deer Stalking

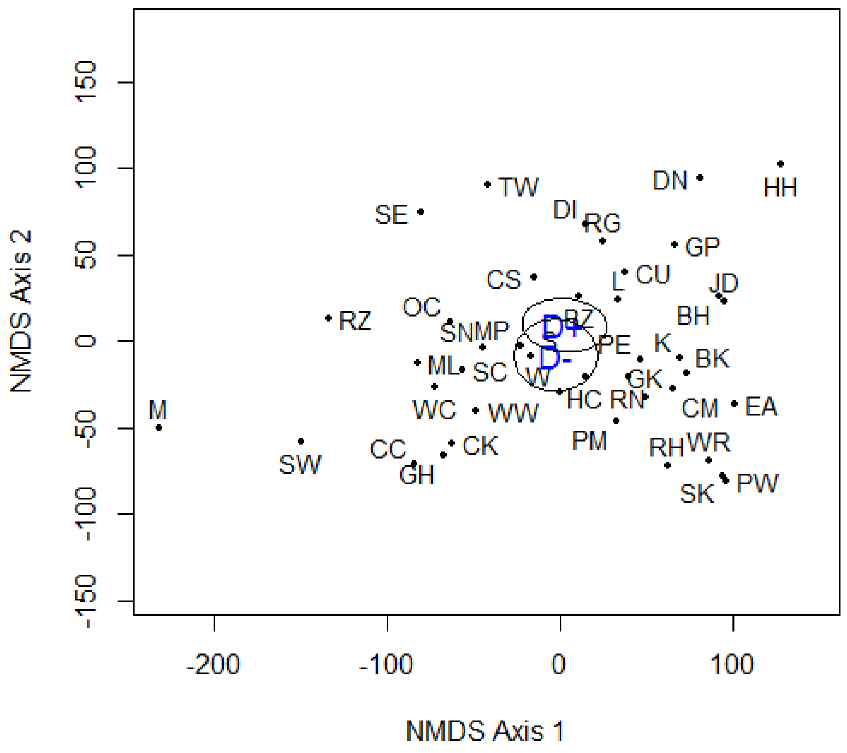

k)Euclidean Distance:Sheep Grazing

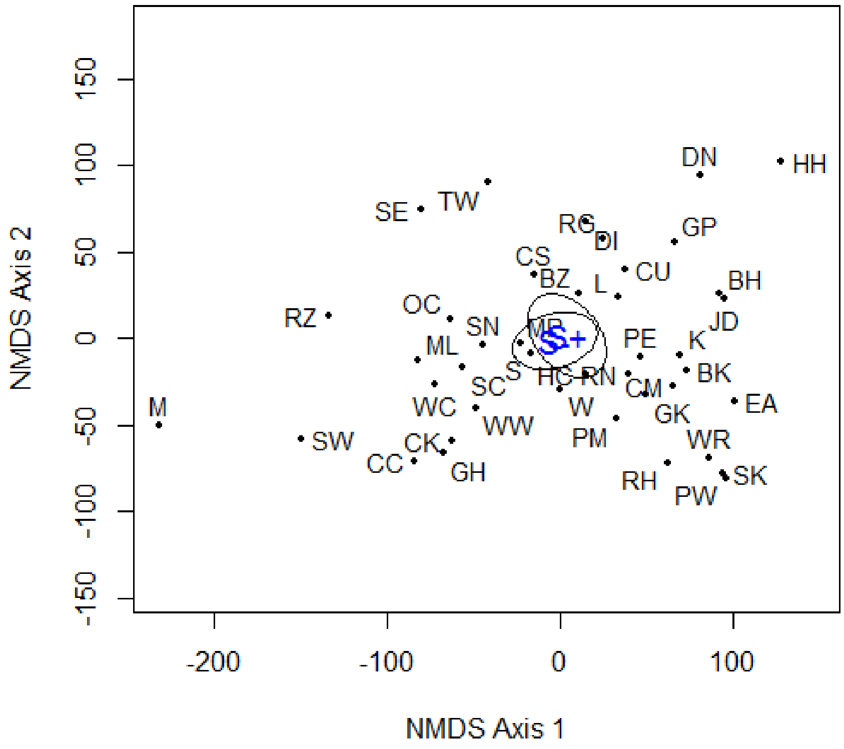

l)Euclidean Distance:APC & MPC

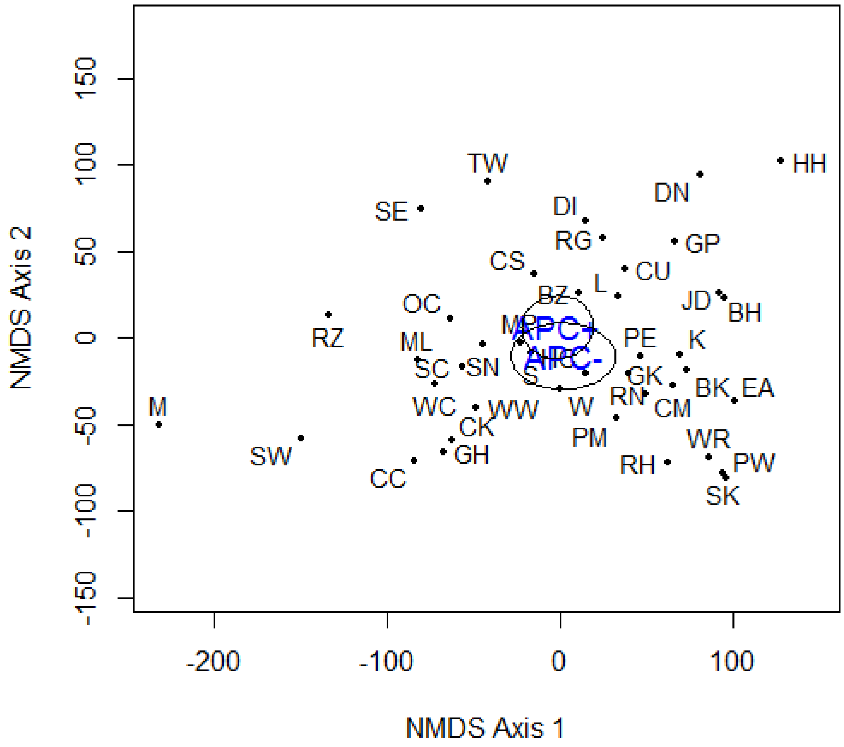

Bray-Curtis Dissimilarity NMDS Ordinations Plots

m) Bray-Curtis: Continous Variables

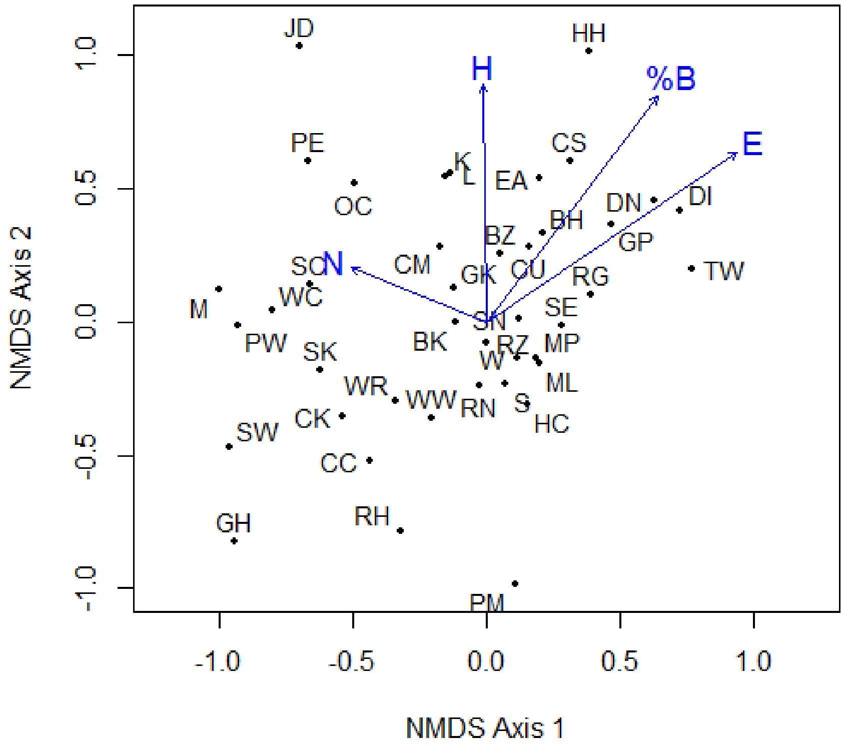

n) Bray-Curtis: Grouse Shooting

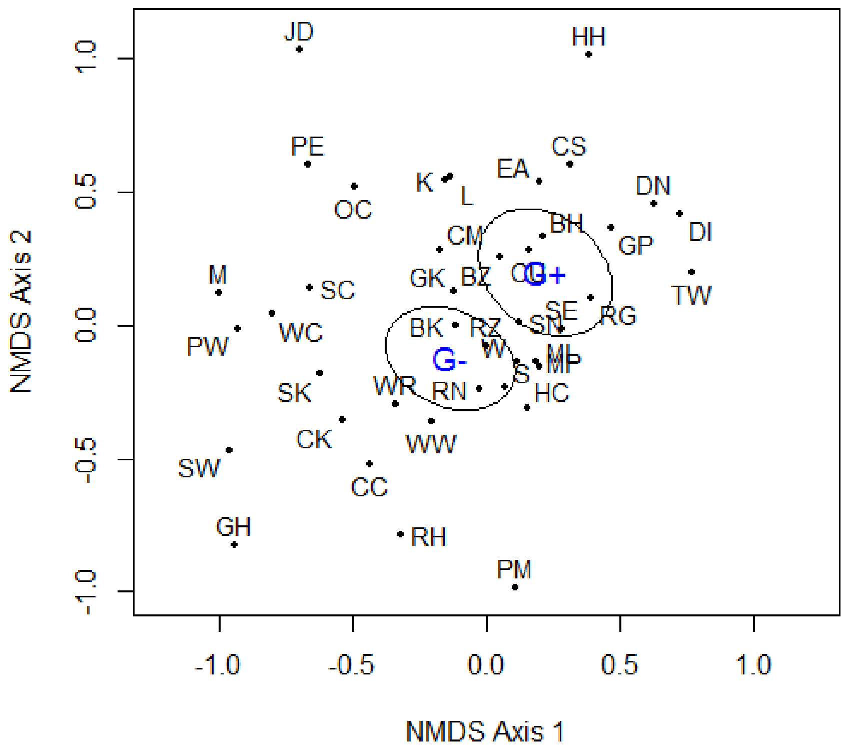

o)Bray-Curtis: Conservation

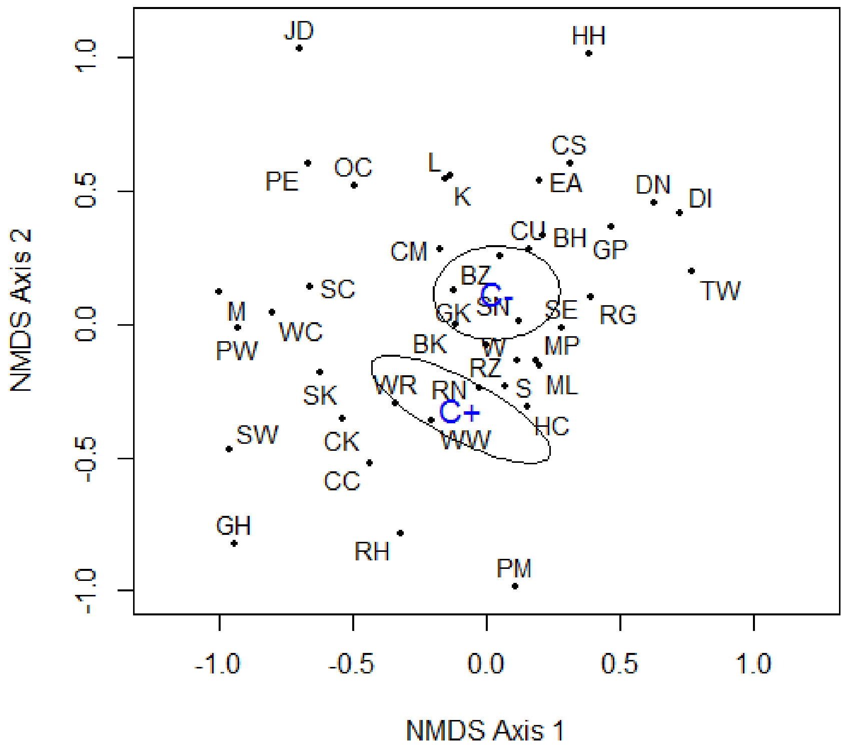

p)Bray-Curtis: Deer Stalking

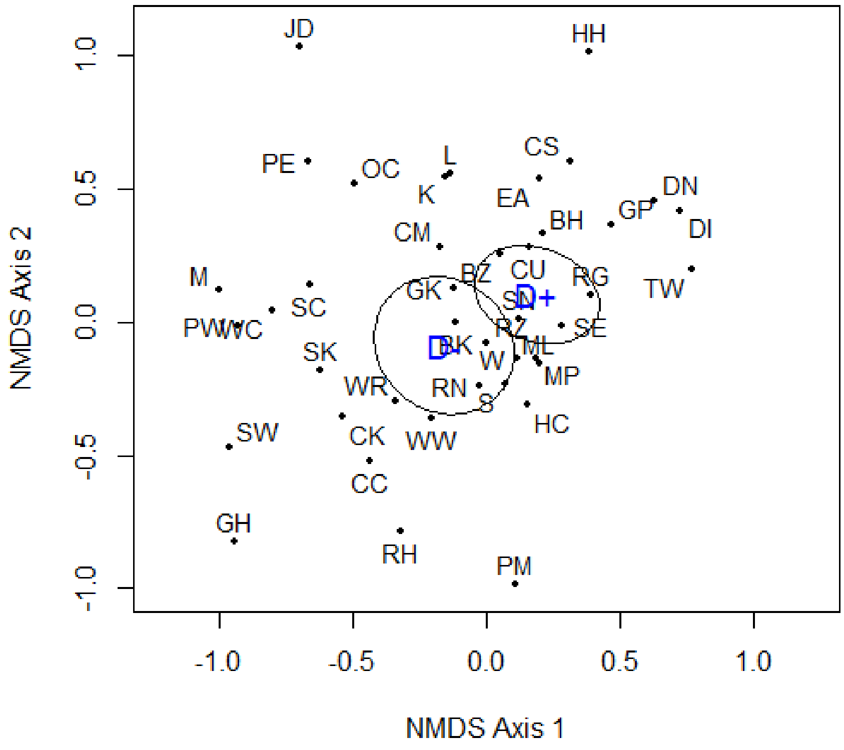

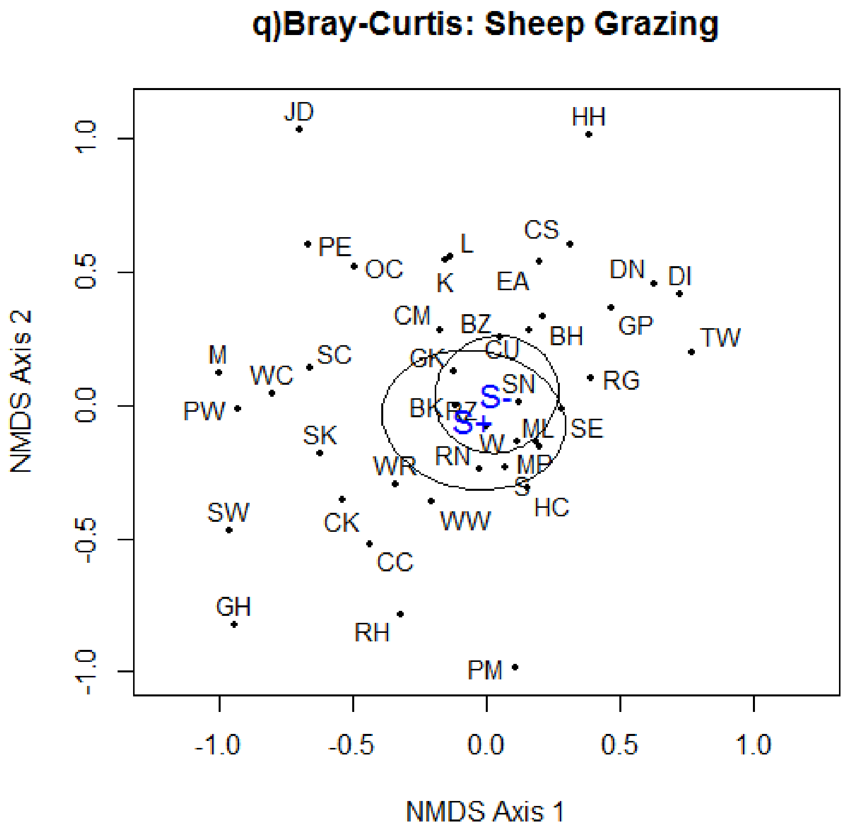

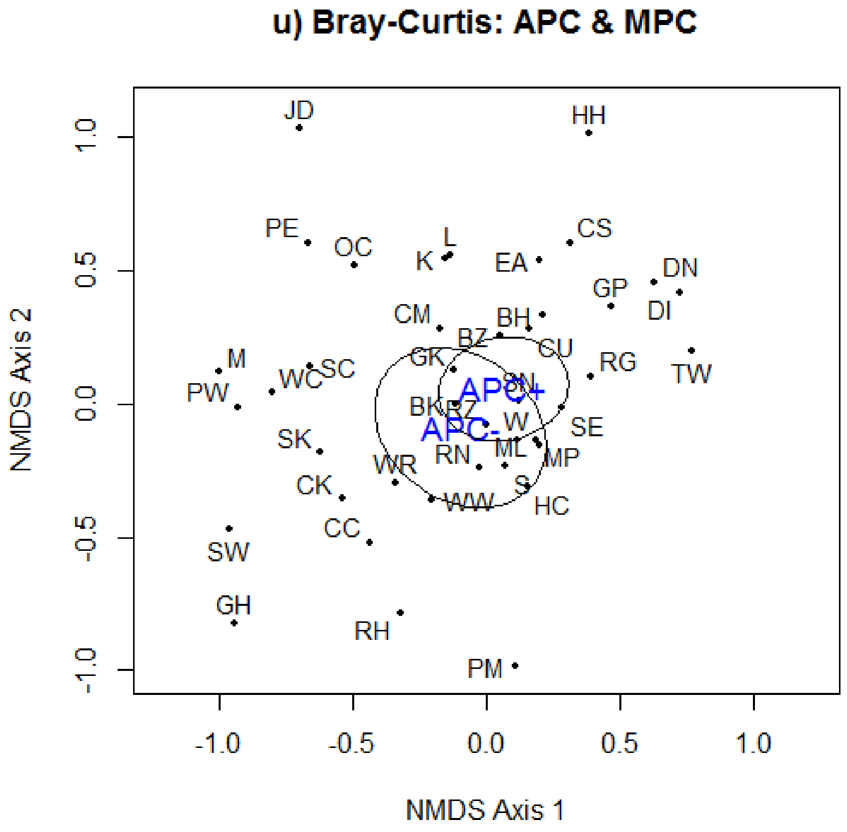

S3 Fig. Enlarged Ordination plots of Raup-Crick (a-f), Euclindean Distance g-l), and Bray Curtis Dissimilarity (m-u) with 95% Standard Error centroids showing species associated with dominant management and management activities (e.g. G+ denotes estates managed for grouse and G- indicates the rest of the estates). Species are coded following British Trust for Ornithology survey codes; Mallard - MA; Tufted Duck - TU; Red Grouse - RG; Ptarmigan - PM; Black Grouse - BK; Pheasant - PH; Red-throated

Diver - RH; Cormorant - CA; Grey Heron - H.; Hen Harrier - HH; Buzzard - BZ; Golden Eagle - EA; Kestrel - K.; Merlin - ML; Peregrine - PE; Moorhen - MH; Oystercatcher - OC; Golden Plover - GP; Lapwing - L.; Dunlin - DN; Snipe - SN; Curlew - CU; Greenshank - GK; Common Sandpiper - CS; Black-headed Gull - BH; Common Gull - CM; Lesser Black-backed Gull - LB; Herring Gull - HG; Greater Black-backed Gull - GB; Woodpigeon - WP; Cuckoo - CK; Short-eared Owl - SE; Swallow - SL; Meadow Pipit - MP; Pied Wagtail - PW; Dipper - DI; Dunnock - D; Robin - R.; Whinchat - WC; Stonechat - SC; Wheatear - W.; Ring Ouzel - RZ; Skylark - S.; House Martin - HM; Wren - WR; Mistle Thrush - M.; Grasshopper Warbler - GH; Sedge Warbler - SW; Chiffchaff - CC; Willow Warbler - WW; Great Tit - GT; Jackdaw - JD; Carrion/Hooded Crow - HC.; Raven - RN; Chaffinch - CH; Greenfinch - GR; Siskin - SK; Twite – TW
