# Supplementary material for: Impact of Management on Avian Communities in the Scottish Highlands
Source: PLoS One. 2016 May 19;11(5):e0155473. doi: 10.1371/journal.pone.0155473 (PMC4873258; doi:10.1371/journal.pone.0155473)
Supplement: S1 Table — Alphabetical list of all bird species recorded during surveys and indication of whether a species is included in the ordination plots (all species were included in the analysis, but for clarity not all species are shown in Fig 2). (DOCX) [file pone.0155473.s003.docx]

**Supplementary Information. S1 Table**. Alphabetical list of all bird species recorded during surveys and indication of whether a species is included in the ordination plots (all species were included in the analysis, but for clarity not all species are shown in Figure 1).

| Scientific Name | Common Name | Shown in ordination plots |
| --- | --- | --- |
| *Acrocephalus schoenobaenus* | Sedge warbler | Yes |
| *Actitis hypoleucos* | Common sandpiper | Yes |
| *Alauda arvensis* | Skylark | Yes |
| *Anas platyrhynchos* | Mallard |  |
| *Anthus pratensis* | Meadow pipit | Yes |
| *Aquila chrysaetos* | Golden eagle | Yes |
| *Ardea cinerea* | Grey Heron |  |
| *Asio flammeus* | Short-eared owl | Yes |
| *Aythya fuligula* | Tufted Duck |  |
| *Buteo buteo* | Buzzard | Yes |
| *Calidris alpina* | Dunlin | Yes |
| *Carduelis chloris* | Greenfinch |  |
| *Carduelis flavirostris* | Twite | Yes |
| *Carduelis spinus* | Siskin | Yes |
| *Chroicocephalus ridibundus* | Black-headed gull | Yes |
| *Cinclus cinclus* | Dipper | Yes |
| *Circus cyaneu* | Hen harrier | Yes |
| *Corvus corax* | Raven | Yes |
| *Corvus corona/cornix* | Carrion/hooded crow | Yes |
| *Corvus monedula* | Jackdaw | Yes |
| *Cuculus canorus* | Cuckoo | Yes |
| *Delichon urbica* | House martin |  |
| *Erithacus rubecula* | Robin |  |
| *Falco columbarius* | Merlin | Yes |
| *Falco peregrinus* | Peregrine | Yes |
| *Falco tinnunculus* | Kestrel | Yes |
| *Fringilla coelebs* | Chaffinch |  |
| *Gallinago gallinago* | Common snipe | Yes |
| *Gallinula chloropus* | Moorhen |  |
| *Gavia stellata* | Red-throated diver | Yes |
| *Haematopus ostralegus* | Oyster catcher | Yes |
| *Hirundo rustica* | Swallow |  |
| *Lagopus lagopus* | Red grouse | Yes |
| *Lagopus mutus* | Ptarmigan | Yes |
| *Larus argentatus* | Herrring gull |  |
| *Larus canus* | Common gull | Yes |
| *Larus fuscus* | Lesser black backed gull |  |
| *Larus marinus* | Great black backed gull |  |
| *Locustella naevia* | Grasshopper warbler | Yes |
| *Motacilla alba* | Pied wagtail | Yes |
| *Numenius arquata* | Curlew | Yes |
| *Oenanthe oenanthe* | Wheatear | Yes |
| *Parus major* | Great tit |  |
| *Phalacrocorax spp* | Cormorant |  |
| *Phasianus colchicus* | Pheasant |  |
| *Phylloscopus collybita* | Chiffchaff | Yes |
| *Phylloscopus trochilus* | Willow warbler | Yes |
| *Pigeon* | Pigeon |  |
| *Pluvialis apricaria* | Golden plover | Yes |
| *Prunella modularis* | Dunnock |  |
| *Saxicola rubetra* | Winchat | Yes |
| *Saxicola torquata* | Stonechat | Yes |
| *Tetrao tetrix* | Black grouse | Yes |
| *Tringa nebularia* | Greenshank | Yes |
| *Troglodytes troglodytes* | Wren | Yes |
| *Turdus torquatus* | Ring ouzel | Yes |
| *Turdus viscivorus* | Mistle thrush | Yes |
| *Vanellus vanellus* | Lapwing | Yes |
